# Shear Wave Elastography for Distinguishing Cervical Lymph Node Malignancy: A Prospective, Observational Study

**DOI:** 10.3390/biomedicines13082001

**Published:** 2025-08-18

**Authors:** Dragos A. Termure, Manuela Lenghel, Mindra E. Badea, Horatiu A. Rotar, Ciprian Tomuleasa, Bobe Petrushev, Emil Botan, Madalina Moldovan-Lazar, Alexandru F. Badea

**Affiliations:** 1 Department of Maxillofacial Surgery and Radiology, Iuliu Hatieganu University of Medicine and Pharmacy, 400001 Cluj-Napoca, Romania; dr.horatiu.rotar@gmail.com (H.A.R.); madilazar@yahoo.com (M.M.-L.); 2 Department of Radiology, Iuliu Hatieganu University of Medicine and Pharmacy, 400124 Cluj-Napoca, Romania; lenghel.manuela@gmail.com; 3 Department of Preventive Dentistry, Iuliu Hatieganu University of Medicine and Pharmacy, 400001 Cluj-Napoca, Romania; mindrabadea@gmail.com; 4 Department of Hematology, Iuliu Hatieganu University of Medicine and Pharmacy, 400337 Cluj-Napoca, Romania; ciprian.tomuleasa@gmail.com; 5 Department of Hematology, Ion Chiricuta Clinical Cancer Center, 400124 Cluj-Napoca, Romania; 6 Department of Pathology, Octavian Fodor Regional Institute of Gastroenterology and Hepatology, 400139 Cluj-Napoca, Romania; bobe.petrushev@gmail.com; 7 Department of Pathology, Cluj-Napoca County Clinical Emergency Hospital, 400006 Cluj-Napoca, Romania; emilbotan@gmail.com; 8 Department of Anatomy and Embryology, Iuliu Hatieganu University of Medicine and Pharmacy, 400006 Cluj-Napoca, Romania; alexandru.badea@umfcluj.ro

**Keywords:** shear wave elastography, cervical, lymph node, lymphoma, metastasis

## Abstract

**Simple Summary:**

Accurate and timely diagnosis of cervical lymphadenopathy remains a cornerstone in the management of head and neck pathologies. The aim of our prospective, observational study was to assesses the diagnostic value of shear wave elastography (SWE) for cervical lymph nodes in diagnosing lymphadenopathy. In 35 participants with malignant lymph nodes, we determined that minimal kPa values can be used to differentiate benign lymph nodes from lymphadenopathy due to lymphoma or metastasis. We also saw that each 1 kPa increase in measurement is associated with a 3% increased risk for the lymphadenopathy to be of metastatic origin. Overall, our results show the utility of SWE for the preoperative assessment of lymph nodes in patients with lymphoma or metastasis.

**Abstract:**

**Background/Objectives**: Differentiating between benign and malignant cervical lymph nodes (LNs) is a critical challenge in the clinical setting. We assessed the ability of shear wave elastography (SWE) to distinguish between lymphomas and solid tumor metastases presenting as cervical adenopathy. **Methods**: We performed a single-center, prospective, observational study in adults with clinically suspicious cervical lymph nodes. The ultrasound examination included conventional ultrasound and SWE with quantitative assessment (tissue stiffness in kPa). Pathology examination was the definitive confirmation method. Simple univariate binary logistic regression and multiple univariate binary logistic regression were used. **Results**: The maximum shear wave velocity (SWV) in patients with benign pathologies was 35 kPa, lower than the minimal values for lymphoma (40 kPa) and metastases (50 kPa). Furthermore, squamous cell carcinoma and distant metastases were more prevalent among men. Independent from other factors used in the statistical model, we found a positive association between sex and the presence of metastatic lymph nodes. Finally, each 1 kPa from SWE measurement was associated with a 3% increase in the risk for LNs to present metastatic adenopathy. **Conclusions**: This study highlights the potential of SWE for the preoperative assessment of nodal status in patients with various malignancies affecting the head and neck region, thyroid, and other areas.

## 1. Introduction

The accurate and timely diagnosis of cervical lymphadenopathy remains a cornerstone in the management of head and neck pathologies, including infections, autoimmune disorders, primary malignancies and lymph node (LN) metastasis [1,2]. Multiple primary tumors can have cervical lymph involvement, including salivary glands, thyroid tumors, basal cell carcinoma, and melanoma [1,2], while breast, lung, esophagus, gonad, pancreas, kidney, and bowel tumors have been reported to lead to cervical lymph node metastasis [1,2]. Furthermore, lymphoproliferative diseases (lymphoma/leukemia) are also known to present with lymph node involvement [1,2]. Thus, the intricate task of distinguishing benign from malignant cervical lymph nodes is a critical challenge in the clinical setting, with significant implications for patient management and clinical outcomes.

Shear wave elastography (SWE), less costly than MRI and CT, is an emerging technique to evaluate nodes, including in the neck [3,4]. Although promising, there are no standardized accepted values and knowledge associated with size and shape on CT or MRI is debated. Our study adds to this literature. In this context, shear wave elastography (SWE) has emerged as a promising technique. SWE provides quantitative measurements of tissue stiffness that can help to characterize tissue consistency and identify potential malignancy [5,6]. SWE works by using focused acoustic radiation force impulses to generate transverse (shear) waves in tissue, rather than relying on manual compression as in conventional elastography. The ultrasound system tracks the speed at which these shear waves propagate through the tissue, and from that speed it calculates the Young’s modulus, a quantitative measure of tissue stiffness, expressed in kilopascals (kPa) [7]. Because the process is automated, SWE is operator-independent, reproducible, and free from compression artifacts, making it more reliable than strain-based techniques. The result is a real-time, color-coded elasticity map overlaid on the B-mode image, allowing local stiffness to be measured at any point in the region of interest. Pathologic processes, including inflammation and malignancy, alter the mechanical properties of tissue. In general, malignant lymph nodes tend to be significantly stiffer than both benign nodes and surrounding normal tissues. This increased stiffness is due to tumor cell infiltration, desmoplastic reaction, increased cellular density, and fibrosis. Benign lymph nodes may be firmer than normal tissue (e.g., from reactive hyperplasia) but are usually less stiff than malignant nodes [7].

Building upon this principle, our analysis assesses the diagnostic value of SWE for cervical lymph nodes, examining how this approach can aid in diagnosing lymphadenopathy. Overall, our study included the determination of the role of SWE in classifying lymphomas vs. solid tumor metastases associated with malign adenopathy, as well as demographic and histopathology variables associated with the two etiologies.

## 2. Materials and Methods

### 2.1. Study Design

This single-center, prospective, observational study was conducted to investigate the diagnostic value of SWE in differentiating benign and malignant cervical lymph nodes and in differentiating lymphoma from cervical lymph node metastasis. The study was performed at the Cluj Napoca Emergency County Hospital—Department of Oral and Maxilo-Facial Surgery, Department of Radiology and Imagistics, and Department of Pathology in Cluj Napoca, Romania. The study was conducted in accordance with the Declaration of Helsinki, and approved by the Ethics Committee of “Iuliu Hațieganu” University of Medicine and Pharmacy Cluj Napoca code 117 from 15 April 2021.

Patients were included if they were aged 18 years or older with clinically suspicious cervical lymph nodes identified through physical examination or imaging studies. Patients were referred for the following reasons: enlarged cervical LN with unknown etiology or head and neck squamous cell carcinoma certified through biopsy with enlarged cervical LN at first presentation. All patients with a history of surgically treated head and neck malignancies, prior neck surgery or irradiation, chemotherapy and history of cervical LN biopsy regardless of technique, and who refused the proposed elastography measurement and surgical protocol were excluded from the study.

All eligible participants underwent comprehensive clinical evaluation, including the collection of detailed medical history, physical examination, and imaging studies such as ultrasound, CT, or MRI of the head, neck, chest, or abdomen, depending on the specific disease. Subsequently, SWE examinations of the suspicious cervical lymph nodes were performed. Our reference standard was the surgical pathology result after core biopsy, with excisional biopsy or neck dissection performed for definitive diagnosis.

All of the LNs in our study underwent pathology examination. The surgical technique used for each patient (Table 1) was adapted to the specific disease of the patient, and the treatment protocol was adapted for the specific disease and location and dimensions of the cervical lymphadenopathy.

### 2.2. SWE Measurements

Conventional ultrasound examinations and shear wave elastography were performed with a MACH™ 20 SuperSonic™ Ultrasound System (SuperSonic Imagine, Aixen-Provence, France) with a 7–18 MHz compact linear array transducer. All measurements were carried out by a single practitioner with more than 15 years of experience. The ultrasound examination included conventional ultrasound features, Doppler ultrasound, and SWE with quantitative assessment (tissue stiffness measured in kPa). Conventional ultrasound features of the lesions were the size of the LN, measured in all three perpendicular axes, delineation (clear margins or blurred contour), shape (round or oval), presence/absence of the hilum, and presence of internal changes (microcalcifications and cystic areas). The LNs were classified into two groups, round and oval, based on their axis ratio, defined as the shortest diameter divided by the longest diameter.

Following the Doppler assessment, we stored the following data: central, hilar/peripheral, or mixed vascularity (both central and peripheral). The SWE values of the stiffness of a selected region of interest (ROI) were stored in the database. A 2D SWE elastography color map was displayed on the screen, and ROI boxes were placed within the lesion, trying to encompass the affected LN, but avoiding the necrotic and calcified areas. Lesion stiffness was defined as the mean shear wave velocity (SWV) of five measurements, with this mean value used for statistical analysis. All images were stored digitally. The elastography exam was the last part of the US exam before LN biopsy.

### 2.3. LN Biopsy

Core ultrasound-guided and non-ultrasound-guided biopsy were used for fourteen patients in instances considered suitable, like bulky LNs, with superficial locations and facile access for the biopsy through cut needle, and when the suspected diseases were lymphoma, infection, or inflammation. This procedure was conducted under local anesthesia. The core biopsy procedure provides a large and more intact tissue sample (Supplementary Figure S1), allowing for a comprehensive histological examination and accurate diagnosis.

Excisional lymph node biopsy was performed on five patients in cases when the only lymph nodes considered relevant for the pathological report and thus for a definitive diagnosis were small and located deep in the deep cervical spaces (Supplementary Figure S2). The procedure was conducted under local anesthesia with or without or i.v. sedation or general anesthesia and an LN as a whole was excised (Supplementary Figures S3–S5).

Neck dissection (Supplementary Figure S6) was performed in all patients with a proven head and neck primary localization squamous cell carcinoma, meaning fifty-one patients (Supplementary Figure S7). The neck dissection was performed encompassing different levels of the neck, unilateral or bilateral, depending on the tumor location, stage, and the presence or absence of the clinically proven cervical lymph node metastasis. All of the surgical resection pieces representing cervical lymph node tissue contained the lymph nodes selected and evaluated by SWE prior to surgery (Supplementary Figure S8).

### 2.4. Statistical Analysis

The distribution of continuous variables was evaluated using the Shapiro–Wilk test, along with descriptive measures of skewness and kurtosis, and visual inspection of Q–Q plots. Variables that followed a normal distribution were reported as mean ± standard deviation (SD) and compared using Welch’s *t*-test (for two-group comparisons) or one-way ANOVA (for comparisons between three groups). The assumption of homogeneity of variances was assessed using Levene’s test. Where ANOVA showed significant results, Tukey’s test was used for post-hoc pairwise comparisons.

Categorical variables were summarized as frequencies and percentages. Fisher’s exact test was used to compare proportions between groups when expected cell counts were below 5. Pearson’s Chi-squared test was applied when assumptions were met. Adjustments were made where necessary to ensure statistical accuracy, particularly in subgroup comparisons with small sample sizes.

We used simple univariate binary logistic regression with the dependent variable as a binary (0 corresponding to a lymphoma etiology and 1 corresponding to a metastatic etiology), while the independent variable was SWE assessed in kPa. We then built a multivariable binary logistic regression model, including predictors that were statistically significant in the univariate analysis. These included SWE (kPa), sex, and long diameter (mm). Backward elimination was used for variable selection. The diagnostic performance of the model was evaluated using a receiver operating characteristic (ROC) curve, with the area under the curve (AUC) serving as a measure of discrimination. The optimal SWE cut-off value was determined using the Youden index. Model robustness was assessed through leave-one-out cross-validation (LOOCV). A post-hoc power analysis was performed using G*Power v3.1.9.7, based on the final multivariate logistic regression model including three predictors (SWE, sex, and long diameter). Assuming a medium-to-large effect size (f^2^ = 0.25), α = 0.05, and a total sample size of 41, the estimated statistical power was 72.3%.

The α significance level in the study was 0.05, while *p*-values smaller than 0.05 were considered statistically significant. All analyses were carried out using R version 4.4.1 (Vienna, Austria) [8].

## 3. Results

### 3.1. Participants

During a 3-year time span, 70 consecutive patients presenting in our department with cervical lymphadenopathy were considered for inclusion. None of the patients who were screened were excluded and patients were referred for the following reasons: enlarged cervical LN with unknown etiology (41 LNs) or head and neck squamous cell carcinoma certified through biopsy with enlarged cervical LN at first presentation (39 LNs).

Among the 35 participants with malignant lymph nodes, n = 12 patients presented with lymphoma (14 LNs), n = 4 patients with mixed cellularity Hodgkin lymphoma, n = 4 patients with nodular sclerosing Hodgkin lymphoma, n = 2 patients with diffuse large B-cell lymphoma, n = 1 patient with primary cutaneous follicle center lymphoma with lymph node involvement, and n = 1 patient lymphoblastic B-cell lymphoma. Among the 23 patients with metastasis (27 LNs), the primary tumor type was n = 15 oral squamous cell carcinoma, n = 2 skin squamous cell carcinoma, and n = 1 each of testicular mixed germ cell tumor, prostate adenocarcinoma, papillary thyroid carcinoma, and unknown primary non-keratinizing squamous cell carcinoma.

Demographics and characteristics for participants in the study are shown in Table 2. SWE assessment was performed on n = 39 benign lymph nodes, n = 14 LNs in patients with lymphoma, and n = 27 LNs in patients with metastatic tumors.

### 3.2. Analysis by Lymph Node Type and Constructing the Final Model

**Figure 1:**
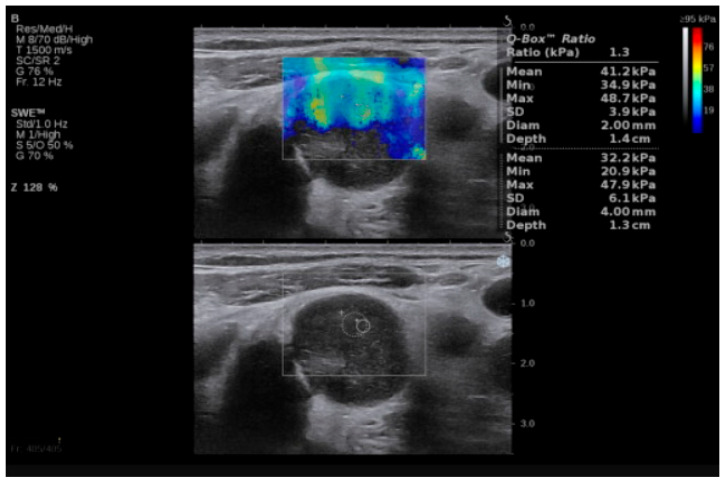
SWE of an LN in a 55-year-old patient with diffuse large B-cell lymphoma. LN, lymph node; SWE, shear wave elastography.

**Figure 2:**
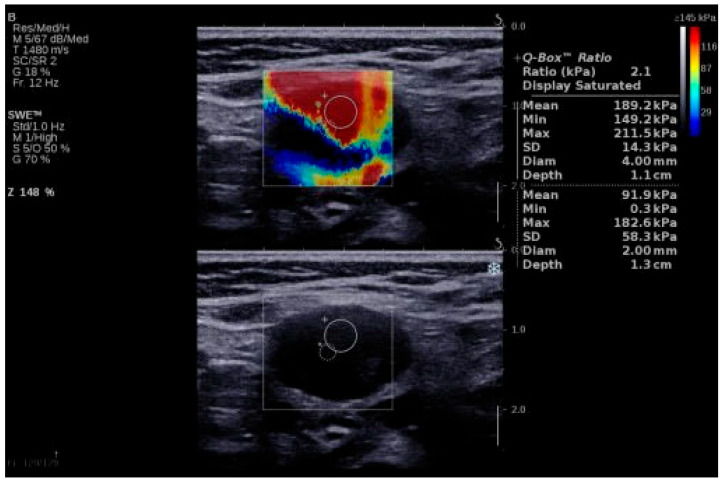
SWE of an LN in a 64-year-old patient with LN metastasis from a squamous cell carcinoma of the anterior floor of the mouth. LN, lymph node; SWE, shear wave elastography.

Examples of SWE analyses of malignant lymph nodes are provided in Figure 1 and Figure 2, and descriptive statistics of SWE by histopathology type (participants with benign cervical lymph nodes, participants with lymphoma and cervical lymphadenopathy, and participants with cervical lymph node metastasis of solid tumors) are shown in Table 3. The maximum SWE value for patients with benign pathologies was 35 kPa, which is lower than both minimal values for lymphoma (40 kPa) and metastases (50 kPa). These results suggest that SWE values under 40 kPa are associated with benign cervical lymph nodes. Given the differences between the benign and malignant lymph nodes highlighted by the SWV, we further concentrated our attention on the differences in tissue stiffness that can be seen in the malignant group and the capacity of SWE in differentiating between the metastatic and lymphoma lymph nodes.

One-way ANOVA showed statistically significant differences in SWE measurements between lymph node types (F = 40.1, *p* < 0.001), so there is a significant difference between the mean SWE values among the three populations.

Table 4 presents the results of the post-hoc analysis using Tukey’s test. Compared to benign lymph nodes, SWE values were significantly higher in both the lymphoma group (mean difference = −52.9 kPa, *p* = 0.001) and the metastasis group (mean difference = −105.1 kPa, *p* < 0.001). Furthermore, SWE values in metastatic lymph nodes were also significantly higher than in lymphomas (mean difference = −52.2 kPa, *p* = 0.003).

These findings support the diagnostic utility of SWE in differentiating between lymph node types. Based on the significant differences between lymphoma and metastasis, we developed a logistic regression model to distinguish these two malignant etiologies.

### 3.3. Final Model Findings

To assess factors associated with metastatic versus lymphomatous cervical lymphadenopathy, we analyzed demographic, ultrasonographic, and elastographic variables (Table 5). There was a statistically significant association between sex and malignancy type (*p* = 0.017). In our cohort, 81% of patients with metastatic cervical lymph nodes were male, while 57% of patients with lymphoma were female. The average SWE values differed significantly between lymphoma and metastasis groups (*p* = 0.003).

Several variables (such as shape and necrosis) had low subgroup counts, limiting statistical reliability. To address this, we applied Fisher’s exact test where appropriate and excluded such variables from the multivariate model to avoid overfitting. Only predictors showing both sufficient subgroup size and statistical significance in univariate analysis were included in the final regression model.

Before constructing the multivariable model, we performed a simple univariate binary logistic regression to examine the isolated predictive value of SWE. This analysis revealed a statistically significant association between SWE and metastatic etiology: for every 1 kPa increase in SWE, the odds of a lymph node being metastatic increased by 3% (OR = 1.03; 95% CI: [1.01; 1.07]; *p* = 0.03), as shown in Table 6.

Using a backward selection algorithm, a reduced model was derived, retaining only predictors that remained independently and statistically significant. All predictors included in the final model demonstrated a significant contribution to distinguishing between metastatic and lymphomatous lymph nodes (Table 7).

In the final model, three variables were retained: sex, SWE (kPa), and long-axis diameter. Male sex was significantly associated with metastasis, with an odds ratio (OR) of 23.2 (95% CI: [3.01, 318], *p* = 0.006), indicating substantially higher odds compared to females. Long-axis diameter showed an inverse relationship with metastasis, where each 1 mm increase was associated with a 6% decrease in the odds of a metastatic diagnosis (OR = 0.94, 95% CI: [0.89, 0.99], *p* = 0.020). Elongated nodes, as opposed to spherical ones, are more likely benign—this has been shown on CT and MRI. Regarding elastographic parameters, SWE remained an independent predictor of metastatic lymphadenopathy: each 1 kPa increase was associated with a 3% increase in the odds of metastasis (OR = 1.03, 95% CI: [1.01, 1.07], *p* = 0.050).

#### Model Performance and Validation

To provide a comparative perspective, we also assessed the diagnostic performance of SWE as a standalone predictor using logistic regression. On the training dataset, the SWE-only model achieved an accuracy of 78.00%, with a Cohen’s kappa coefficient of 0.44 and an AUC of 0.763. Under leave-one-out cross-validation (LOOCV), its performance slightly decreased to an accuracy of 75.60%, a Cohen’s kappa of 0.42, and an AUC of 0.694. The confusion matrices for the SWE-only model under raw and LOOCV conditions are presented in Table 7 and Table 8, respectively.

**Figure 3:**
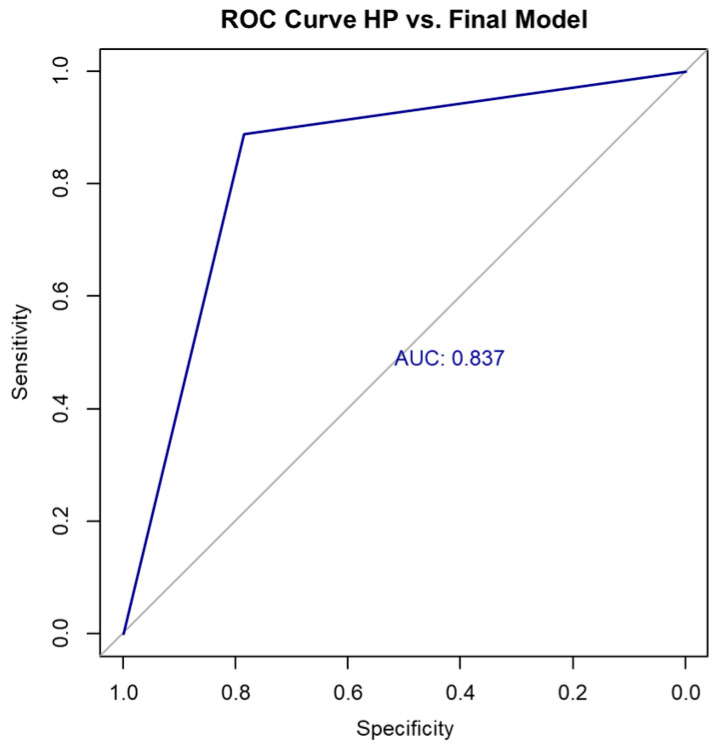
ROC curve HP vs. final model. HP, histopathology; ROC, receiver operating characteristic.

In contrast, the final multivariable logistic regression model—incorporating SWE, sex, and long-axis diameter—demonstrated superior performance compared to the SWE-only model. It achieved an accuracy of 85.36% on the full dataset, with a Cohen’s kappa coefficient of 0.67 (indicating substantial agreement) and an AUC of 0.837, suggesting strong discriminative ability (Figure 3). The confusion matrix for the full dataset is shown in Table 9 and Table 10.

Based on the confusion matrix and the SWE cutoff value of 71.5 kPa (determined using the Youden index), the diagnostic performance of the final model was as follows: sensitivity 88.9%, specificity 78.6%, positive predictive value (PPV) 88.9%, and negative predictive value (NPV) 78.6%. These values reflect the model’s ability to accurately distinguish between metastatic and lymphomatous lymph nodes in our cohort shown in Table 11. 

To evaluate the model’s generalizability, we performed LOOCV. The resulting confusion matrix is shown in Table 12.

**Figure 4:**
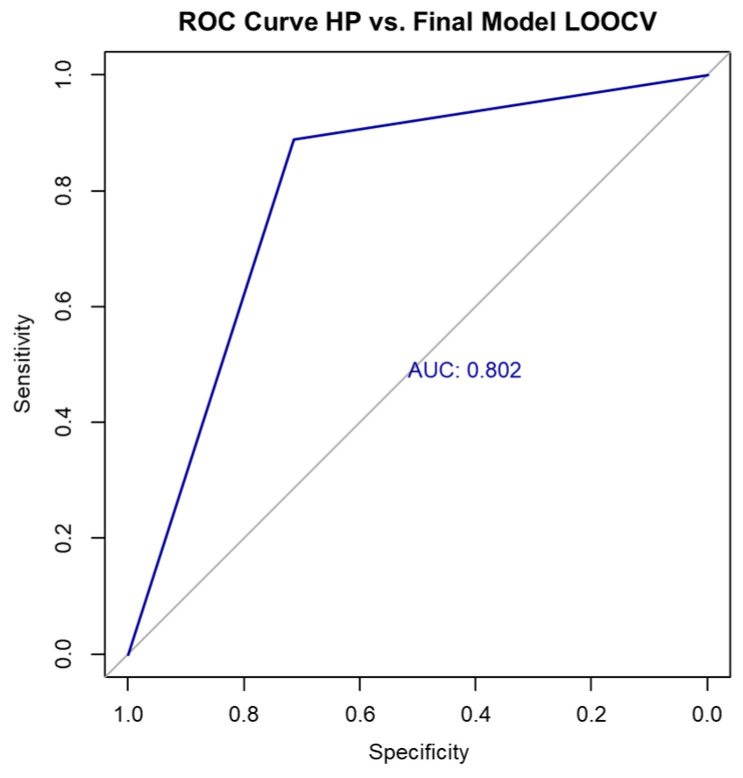
ROC curve for final model performance using LOOCV.

## 4. Discussion

SWE is an emerging imaging modality in the evaluation of lymph nodes. Measuring tissue stiffness is an advance over semiquantitative measures in strain elastography. Metastases are stiffer than lymphoma, and lymphoma is stiffer than non-malignant nodes. The reported differences are not standardized however. Our results indicated the effectiveness of SWE in differentiating between benign and malignant cervical LNs and between metastatic and lymphoma cervical LNs. These diagnostic metrics demonstrate that the model performs well in differentiating between metastatic and lymphomatous cervical lymph nodes. The sensitivity of 78.6% indicates that the model has a good ability to correctly identify patients with metastatic adenopathy. The specificity of 88.9% indicates that the model was highly accurate in correctly identifying patients without metastasis, minimizing false positives. The positive predictive value (PPV) of 78.6% suggests that when the model predicts metastasis, it is correct in a high proportion of cases. Likewise, the negative predictive value (NPV) of 88.9% shows strong reliability in ruling out metastasis when the prediction is lymphoma. An accuracy of 85.36% reflects the overall proportion of correct classifications, while the AUC of 0.837 confirms good discriminative ability of the model. The cutoff value of 71.5 kPa for SWE represents the threshold at which the model optimally differentiates between the two malignancy types. In recent years, SWE has emerged as a promising imaging modality for predicting malignancy in cervical LNs [9,10,11,12,13,14] and an absolute quantification of tissue stiffness, marking a significant advancement over strain elastography, which only provides semiquantitative estimates related to relative tissue strain [15]. SWE is increasingly used as an adjunct to conventional ultrasound for the non-invasive assessment of tissue stiffness, which helps differentiate benign from malignant lesions in organs such as the liver, thyroid, breast, and lymph nodes. In the context of cervical lymph nodes, SWE adds objective quantitative information to the morphologic criteria obtained by B-mode US, improving diagnostic confidence and helping stratify the risk of malignancy. In clinical practice, a low SWE value, indicating low tissue stiffness, suggests benign pathology. If the lymph node also has benign features on B-mode US (such as preserved hilum, oval shape, and normal vascular pattern) and there is no strong clinical suspicion for malignancy, then the combined imaging findings may support a decision to defer immediate biopsy. Instead, the patient may be monitored with follow-up imaging and clinical exams. The decision to skip biopsy based on low SWE values must always consider the full clinical context, including patient history, risk factors, and other sonographic features to avoid false negatives. SWE alone should not override suspicious clinical findings. ARFI and SSI are shear wave-based elastography techniques and both techniques are based on an acoustic radiation force used to generate a shear wave. The differences between ARFI and SSI are in the frame rates required to acquire images and in the strength of the acoustic radiation force [16].

The main promise of elastography in predicting the biological nature of a lesion has been based on the premise that malignant tumors have higher stiffness than benign tumors [9]. Malignant LNs have a tendency to show higher tissue stiffness and this is due to the associated altered tissue composition and structure. SWE provides an absolute quantification of tissue stiffness without the effort of free-hand compression, which means it is less operator dependent and more reproducible [9,10].

Tumor cells in metastatic lymph nodes typically invade through the afferent lymphatic vessels located at the capsule. These vessels are situated on the convex side opposite the lymph hilum and drain into the subcapsular lymph sinuses beneath the capsule. Consequently, during the early stages of metastasis, part of the cortex is initially affected, resulting in localized thickening of the cortical area. The invasion gradually progresses into the lymph medulla, creating lumps of varying sizes within the medulla, which are intermixed with the surrounding normal lymphatic tissue. As tumor cells proliferate and break through the lymph node capsule, they invade the surrounding connective tissues leading to the clustering of multiple lymph nodes and adhesion to adjacent tissues. Consequently, the affected tissue becomes stiffer, with reduced elasticity and increased SWV values [17,18,19].

In the early stages of lymph node metastasis, tumor cells invade only the cortex of the nodes, leaving most of the surrounding tissue unaffected and with no significant change in their consistency. However, in the later stages, the invasion of tumor cells can cause the entire lymph node to undergo liquefaction; in both cases, the result is tissue softening and a decrease in SWV values [9,20,21]. This process of the tumor cell invasion of the LN and the changes that occur gradually can explain the false positive and false negative results found in the literature. Lymphoma is a type of cancer that arises from lymph nodes or other lymphoid tissues. A histological examination reveals a proliferation of tumor cells in the lymph nodes or surrounding tissues. In advanced stages, the affected tissues may have a relatively hard texture and may exhibit adhesion and fusion with adjacent structures. Research indicates that the SWV is higher in malignant LNs than in their nonmetastatic counterparts [22].

SWE is recommended as an effective imaging technique for diagnosing malignant cervical LNs [23,24,25,26,27,28], with a meta-analysis of eight studies showing a summary sensitivity of 81% and a specificity of 88% for diagnosing malignant cervical lymph nodes with no significant differences in results between ARFI and SSI [29]. SWE appeared as unsuitable for cancer screening in 2012 [15]. However, in 2016, SWE was seen as a promising reproducible quantitative tool with which to predict malignant head and neck LNs, especially sub-centimeter nodes [30], and its accuracy improved over time, although SWE seems to remain a promising investigation when associated with other investigations like conventional ultrasound or diffusion-weighted magnetic resonance imaging (DW-MRI) [31,32,33]. This association of imaging techniques led to an accuracy rate increase from 79.3% to 85.3% [31,32,33].

Various studies report different predictive values for SWE based on selected population and lymph node groups. Our study, in contrast to the study by Herman et al. [10], which reported that lymphoma LNs were not generally stiffer than benign lymph nodes, has uncovered a significant difference in tissue stiffness between these two pathological entities that may affect the cervical LNs. Different results may be due to variations in study design, patient populations, sample size, equipment, operator experience, or the criteria used for image interpretation. For example, one study might include only early-stage cases with small, cortex-confined metastases, while another might include a mix of early and advanced cases with necrosis, leading to differences in measured stiffness value. We found that LNs affected by lymphoma to be significantly stiffer with a minimum SWV of 40 kPa, while the maximum SWV measured for benign cervical LNs was 35 kPa. Overall, our study findings are in line with the report by Chae et al. [9], which demonstrates that SWE can be a valuable tool in distinguishing between the two malignant entities that affect cervical lymph nodes, namely metastasis and lymphoma. Our results showed that the SWV values of metastatic lymph nodes were significantly higher than those of lymphoma, indicating that SWV is effective in distinguishing metastatic from lymphoma cervical LNs. Despite the inclusion of various malignant lymph nodes with different primary malignancies and lymphoma subtypes in our study, SWE still proves beneficial in the differentiation of cervical metastasis lymph nodes and lymphoma.

Furthermore, diagnostic accuracy can be improved when SWE is used in conjunction with conventional ultrasound. Despite the inclusion of various malignant lymph nodes with different primary malignancies and lymphoma subtypes in our study, SWE still proves beneficial in the differentiation of cervical metastasis lymph nodes and lymphoma.

Elastography faces challenges in achieving homogeneous measurements due to biological variability within tissues, such as fibrosis or necrosis, and technical factors like probe pressure and acquisition settings. These inconsistencies complicate stiffness interpretation and hinder the establishment of universal diagnostic cut-offs [5,6].

Our study also has several limitations. Measurements were carried out by one specialist, so we could not assess inter-operator reliability. However, as pointed out by Chae et al., there is a generally high concordance between assessors when employing this technique [9]. A second limitation is related to the relatively small sample size, and the fact that benign LNs included in the study, with one exception, were reactive LNs. Another limitation of our study is the heterogeneity of the malignant lymph node cohort, which included metastases from various primary tumors and multiple lymphoma subtypes. These different pathologies may have distinct stiffness profiles and patterns of lymph node involvement, potentially influencing SWE measurements and complicating the interpretation of pooled results. Therefore, further studies are warranted for determining elastographic parameters in lymph nodes affected by non-malignant pathologies.

## 5. Conclusions

Our study, though small and with different levels of kPa than some other studies, contributes to the data reported by others that SWE may at times be useful in evaluating neck nodes when lymphoma and metastases are in the differential diagnosis.

## Figures and Tables

**Table 1:** Surgical technique paired with patients in the study and the specific disease.

	Patients with Benign Lymph Nodes (N = 35)	Patients with Lymphoma (N = 12)	Patients with Metastasis (N = 23)
Core biopsy	1 (2.85%)	11 (91.66%)	2 (8.69%)
Excisional biopsy	1 (2.85%)	1 (8.33%)	3 (13.04%)
Neck dissection	33 (94.28%)	0 (0%)	18 (78.26%)

**Table 2:** Baseline demographics and characteristics of participants in the study.

	Patients with Benign Lymph Nodes (N = 35)	Patients with Lymphoma (N = 12)	Patients with Metastasis (N = 23)
Average age (min, max),	53 (27, 77)	47 (18, 74)	62 (19, 82)
Sex, n (%)			
Female	20 (57%)	6 (50%)	4 (17%)
Male	15 (43%)	6 (50%)	17 (83%)
SWV, mean (SD)	17.7 (5.42)	70.6 (27.96)	122.8 (89.0)

N, participant numbers; SWV, shear wave velocity; and SD, standard deviation.

**Table 3:** Descriptive statistics of SWV (kPa) measurements by lymph node type.

Histopathology Type	Mean (SD)	Median (Min, Max)
Benign (N = 39)	17.7 (5.42)	18.0 (6.00, 35.0)
Lymphoma (N = 14)	70.6 (27.96)	63.0 (40.00, 115.0)
Metastasis (N = 27)	122.8 (77.40)	89.0 (50.00, 300.0)

N, lymph node number; SD, standard deviation; and SWV, shear wave velocity.

**Table 4:** ANOVA post-hoc comparison by lymph node type.

Comparison	Mean Difference (in kPa)	SE	df	t	p_adjusted *
Benign–Lymphoma	−52.9	14.5	77.0	−3.65	0.001
Benign–Metastasis	−105.1	11.7	77.0	−9.01	<0.001
Lymphoma–Metastasis	−52.2	15.3	77.0	−3.40	0.003

SE, standard error. * The *p*-value was obtained by applying Tukey’s test.

**Table 5:** Descriptive analysis of variables in the study.

	Overall Lymph Nodes (N = 41)	Lymph Nodes in Patients with Lymphoma (N = 14)	Lymph Nodes in Patients with Metastasis (N = 27)	*p*-Value ^1^
Age, mean (SD)	56.80 (16.01)	49.79 (18.75)	60.44 (13.35)	0.073
Sex, n (%)				0.017
Female	13 (32)	8 (57)	5 (19)	
Male	28 (68)	6 (43)	22 (81)	
SWE, mean (SD)	105.01 (69.11)	70.64 (27.96)	122.83 (77.40)	0.003
Shape, n (%)				0.60
Oval	4 (9.8)	2 (14)	2 (7.4)	
Round	37 (90)	12 (86)	25 (93)	
Long diameter, mean (SD)	32.29 (20.18)	39.07 (25.24)	28.78 (16.44)	0.18
SL ratio, mean (SD)	0.68 (0.14)	0.69 (0.16)	0.68 (0.13)	0.88
Necrosis, yes, n (%)	16 (39)	3 (21)	13 (48)	0.10

^1^ A Welch two-sample *t*-test was used for continuous variables; Fisher’s exact test was applied to sex and lymph node shape; and Pearson’s Chi-squared test was used to assess differences in the proportions of categorical variables such as necrosis presence.

**Table 6:** Simple univariate binary logistic regression for SWE.

Predictor	N	Metastatic Etiology N	OR (95% CI) ^1^	*p*-Value
SWE (kPa)	41	27	1.03 (1.01 to 1.07)	0.03

^1^ OR = odds ratio; CI = confidence interval.

**Table 7:** Predictive factors based on a backward selection model.

Predictor	All Pathologic Lymph Nodes (N)	Metastatic Etiology (N)	OR (95% CI)	*p*-Value
Sex				
F	13	5	—	
M	28	22	23.2 (3.01 to 318)	0.006
SWE, kPa	41	27	1.03 (1.01 to 1.07)	0.050
Long diameter	41	27	0.94 (0.89 to 0.99)	0.020

CI, confidence interval; OR, odds ratio; and SWE, shear wave elastography.

**Table 8:** Confusion matrix—SWE-only model (training set).

	Final Model Lymphoma	Final Model Metastases
HP Lymphoma	7	7
HP Metastases	2	25

**Table 9:** Confusion matrix—SWE-only model (LOOCV).

	Final Model Lymphoma	Final Model Metastases
HP Lymphoma	7	7
HP Metastases	3	24

**Table 10:** Confusion matrix—final model (entire cohort).

	Final Model Lymphoma	Final Model Metastases
HP Lymphoma	11	3
HP Metastases	3	24

**Table 11:** Diagnostic performance metrics of the final model (entire cohort).

Metric	Value (%)
Sensitivity	88.9
Specificity	78.6
Positive Predictive Value (PPV)	88.9
Negative Predictive Value (NPV)	78.6

Metrics were calculated based on the optimal SWE cutoff value of 71.5 kPa, determined using the Youden index.

**Table 12:** Confusion matrix of the final model under LOOCV.

	Final Model Lymphoma	Final Model Metastases
HP Lymphoma	10	4
HP Metastases	3	24

Under LOOCV, the model maintained robust performance with an accuracy of 82.92%, a Cohen’s kappa of 0.613, and an AUC of 0.802 (Figure 4), confirming good classification capability even in cross-validated testing.

## Data Availability

The research data can be found in the Cluj Napoca County Hospital data bases and Ion Chiricuta Clinical Cancer Center.

## Supplementary Materials

The following supporting information can be downloaded at https://www.mdpi.com/article/10.3390/biomedicines13082001/s1: Figure S1 Core biopsy; Figure S2 Excisional lymph node biopsy; Figure S3 Clinical image of a patient with mixed cellularity Hodgkin lymphoma; Figure S4 Excisional biopsy; Figure S5 Fully excised lymph node; Figure S6 Left side neck dissection of levels Ia-IV; Figure S7 Clinical image of a patient with left side cervical LN metastasis from an oral squamous cell carcinoma; Figure S8 Unilateral neck dissection specimen en-block with primary tumor of the oral cavity

## References

[1] Agarwal M., Nabavizadeh S.A., Mohan S. (2017). Chapter 6 Non-squamous cell causes of cervical lymphadenopathy. Semin. Ultrasound CT MRI.

[2] Sakr M. (2016). Chapter 8 Cervical: Lymphadenopathy. Head and Neck and Endocrine Surgery.

[3] Chami L., Giron A., Ezziane L., Leblond V., Charlotte F., Pellot-Barakat C., Lucidarme O. (2021). Quantitative and Qualitative Approach for Shear Wave Elastography in Superficial Lymph Nodes. Ultrasound Med. Biol..

[4] Gao Y., Zhao Y., Choi S., Chaurasia A., Ding H., Haroon A., Wan S., Adeleke S. (2022). Evaluating Different Quantitative Shear Wave Parameters of Ultrasound Elastography in the Diagnosis of Lymph Node Malignancies: A Systematic Review and Meta-Analysis. Cancers.

[5] Wang B., Guo Q., Wang J.-Y., Yu Y., Yi A.-J., Cui X.-W., Dietrich C.F. (2021). Ultrasound elastography for the evaluation of lymph nodes. Front. Oncol..

[6] Sigrist R.M.S., Liau J., Kaffas A.E., Chammas M.C., Willmann J.K. (2017). Ultrasound elastography: Review of techniques and clinical applications. Theranostics.

[7] Arda K., Ciledag N., Aktas E., Aribas B.K., Köse K. (2011). Quantitative assessment of normal soft-tissue elasticity using shear-wave ultrasound elastography. Am. J. Roentgenol..

[8] R Core Team (2024). R: A Language and Environment for Statistical Computing.

[9] Sy C., Jung H.N., Ryoo I., Suh S. (2019). Differentiating cervical metastatic lymphadenopathy and lymphoma by shear wave elastography. Sci. Rep..

[10] Heřman J., Sedláčková Z., Fürst T., Vachutka J., Salzman R., Vomáčka J., Heřman M. (2019). The role of ultrasound and shear-wave elastography in evaluation of cervical lymph nodes. BioMed Res. Int..

[11] Sasaki Y., Ogura I. (2019). Shear wave elastography in differentiating between benign and malignant cervical lymph nodes in patients with oral carcinoma. Dentomaxillofacial Radiol..

[12] Azizi G., Keller J.M., Mayo M.L., Piper K., Puett D., Earp K.M., Malchoff C.D. (2016). Shear wave elastography and cervical lymph nodes: Predicting malignancy. Ultrasound Med. Biol..

[13] Kerim A., El Abd A.M., Naguib N.N. (2023). Shear wave elastography versus strain elastography to identify benign superficial lymph nodes: Sonographic assessment with histopathological confirmation. Egypt. J. Radiol. Nucl. Med..

[14] Sun Y.-M., Dong H., Du Z.-Y., Yang Z.-L., Zhao C., Chong J., Li P. (2020). The effect of regions-of-interest and elasticity modulus selection on differentiating benign and malignant cervical lymph nodes with shear wave elastography. Clinics.

[15] Bhatia K.S.S., Cho C.C.M., Tong C.S.L., Yuen E.H.Y., Ahuja A.T. (2012). Shear wave elasticity imaging of cervical lymph nodes. Ultrasound Med. Biol..

[16] Suh C.H., Kim S.Y., Kim K.W., Lim Y.-S., Lee S.J., Lee M.-G., Lee J., Lee S.-G., Yu E. (2014). Determination of normal hepatic elasticity by using real-time shear-wave elastography. Radiology.

[17] Steinkamp H.J., Wissgott C., Rademaker J., Felix R. (2001). Current status of power Doppler and color Doppler sonography in the differential diagnosis of lymph node lesions. Eur. Radiol..

[18] Sakai F., Kiyono K., Sone S., Kondo Y., Oguchi M., Watanabe T., Sakai Y., Imai Y., Takeda S., Yamamoto K. (1988). Ultrasonic evaluation of cervical metastatic lymphadenopathy. J. Ultrasound Med..

[19] Vassallo P., Wernecke K., Roos N., Peters P.E. (1992). Differentiation of benign from malignant superficial lymphadenopathy: The role of high-resolution US. Radiology.

[20] Rosário P.W.S., de Faria S., Bicalho L., Alves M.F.G., Borges M.A.R., Purisch S., Padrão E.L., Rezende L.L., Barroso Á.L. (2005). Ultrasonographic differentiation between metastatic and benign lymph nodes in patients with papillary thyroid carcinoma. J. Ultrasound Med..

[21] Ahuja A.T., Ying M., Ho S.Y., Antonio G., Lee Y.P., King A.D., Wong K.T. (2008). Ultrasound of malignant cervical lymph nodes. Cancer Imaging.

[22] Zhao Y., Xi J., Zhao B., Xiong W., Jiang D., Yang L., Cai Z., Liu T., Jiang H., Rong S. (2017). Preliminary evaluation of virtual touch tissue imaging quantification for differential diagnosis of metastatic and nonmetastatic cervical lymph nodes. J. Ultrasound Med..

[23] Choi Y.J., Lee J.H., Lim H.K., Kim S.Y., Han M.W., Cho K.-J., Baek J.H. (2013). Quantitative shear wave elastography in the evaluation of metastatic cervical lymph nodes. Ultrasound Med. Biol..

[24] Fujiwara T., Tomokuni J., Iwanaga K., Ooba S., Haji T. (2013). Acoustic radiation force impulse imaging for reactive and malignant/metastatic cervical lymph nodes. Ultrasound Med. Biol..

[25] Jung W.S., Kim J.-A., Son E.J., Youk J.H., Park C.S. (2015). Shear wave elastography in evaluation of cervical lymph node metastasis of papillary thyroid carcinoma: Elasticity index as a prognostic implication. Ann. Surg. Oncol..

[26] Meng W., Xing P., Chen Q., Wu C. (2013). Initial experience of acoustic radiation force impulse ultrasound imaging of cervical lymph nodes. Eur. J. Radiol..

[27] Zhang J.-P., Liu H.-Y., Ning C.-P., Chong J., Sun Y.-M. (2015). Quantitative analysis of enlarged cervical lymph nodes with ultrasound elastography. Asian Pac. J. Cancer Prev..

[28] Cheng K.L., Choi Y.J., Shim W.H., Lee J.H., Baek J.H. (2016). Virtual touch tissue imaging quantification shear wave elastography: Prospective assessment of cervical lymph nodes. Ultrasound Med. Biol..

[29] Suh C.H., Choi Y.J., Baek J.H., Lee J.H. (2016). The diagnostic performance of shear wave elastography for malignant cervical lymph nodes: A systematic review and meta-analysis. Eur. Radiol..

[30] Desmots F., Fakhry N., Mancini J., Reyre A., Vidal V., Jacquier A., Santini L., Moulin G., Varoquaux A. (2016). Shear wave elastography in head and neck lymph node assessment: Image quality and diagnostic impact compared with B-mode and Doppler ultrasonography. Ultrasound Med. Biol..

[31] Öztürk V.S., Ertekin E. (2021). Diagnostic performance of shear wave elastography and diffusion-weighted magnetic resonance imaging in cervical lymph nodes: A comparative study. Turk. J. Med. Sci..

[32] Kang H.J., Seo M., Sohn Y.-M., Yun S.J., Min S.Y., You M.-W., Yeon E.K. (2019). Comparison of diagnostic performance of B-mode ultrasonography and shear wave elastography in cervical lymph nodes. Ultrasound Q..

[33] Kılıç A., Er H.Ç. (2019). Virtual touch tissue imaging quantification shear wave elastography for determining benign versus malignant cervical lymph nodes: A comparison with conventional ultrasound. Diagn. Interv. Radiol..

