# Supplementary material for: Shear Wave Elastography for Distinguishing Cervical Lymph Node Malignancy: A Prospective, Observational Study
Source: Biomedicines. 2025 Aug 18;13(8):2001. doi: 10.3390/biomedicines13082001 (PMC12383889; doi:10.3390/biomedicines13082001)

**Supplementary Materials**

**Supplementary Figure S1. Core biopsy**

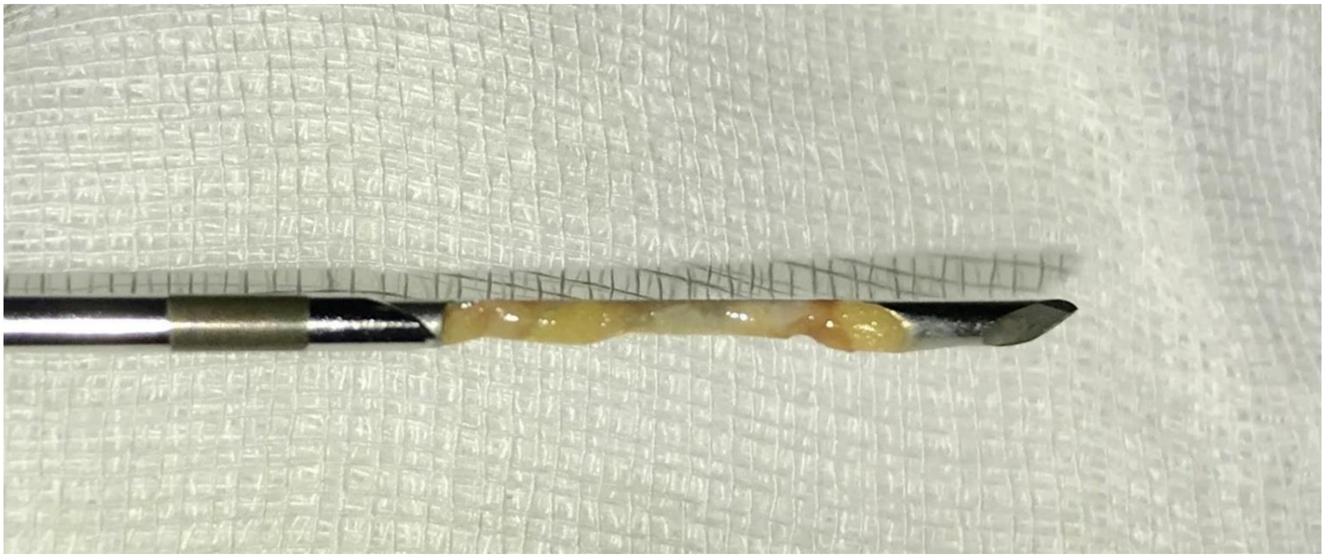

**Supplementary Figure S2. Excisional lymph node biopsy**

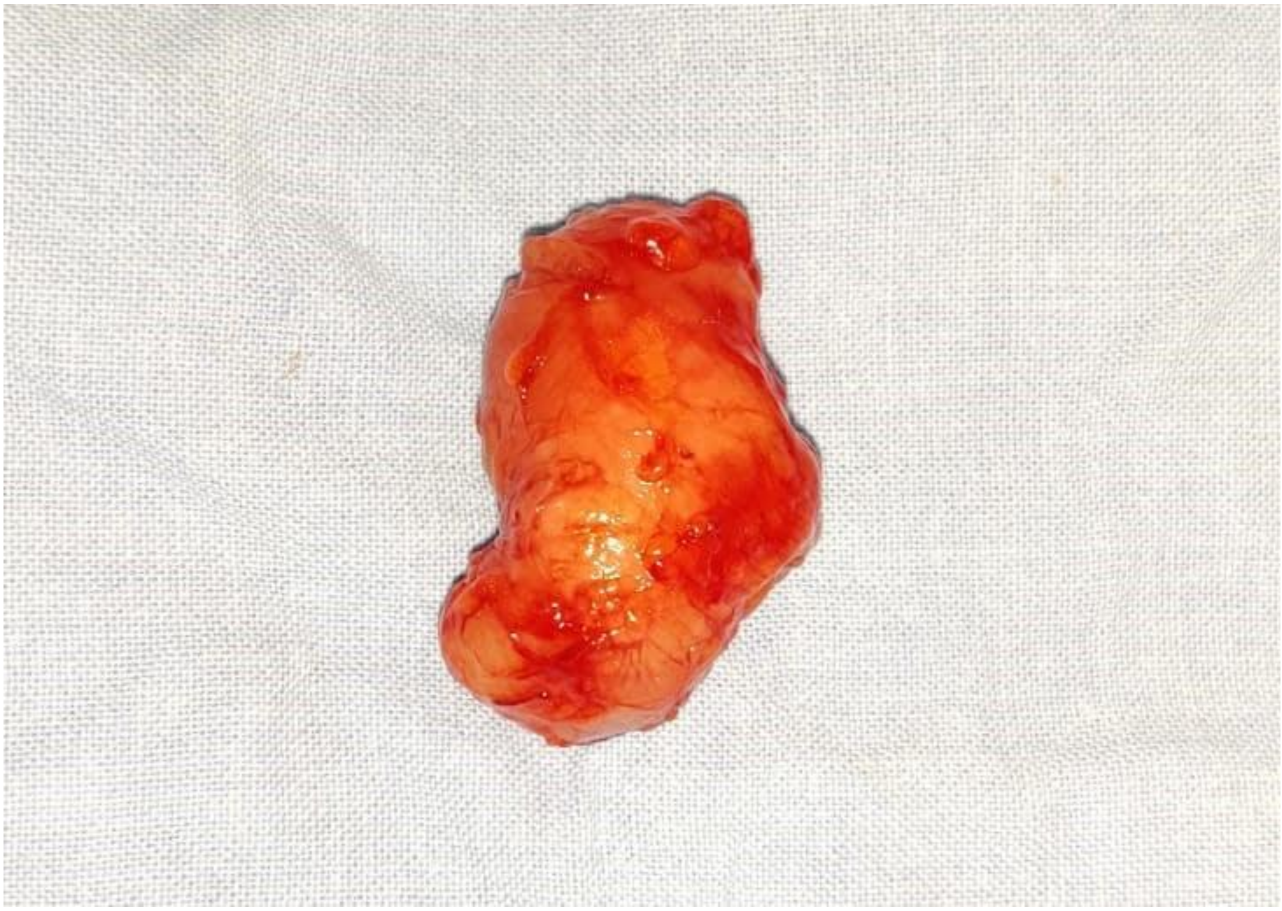

**Supplementary Figure S3. Clinical image of a patient with mixed cellularity Hodgkin lymphoma**

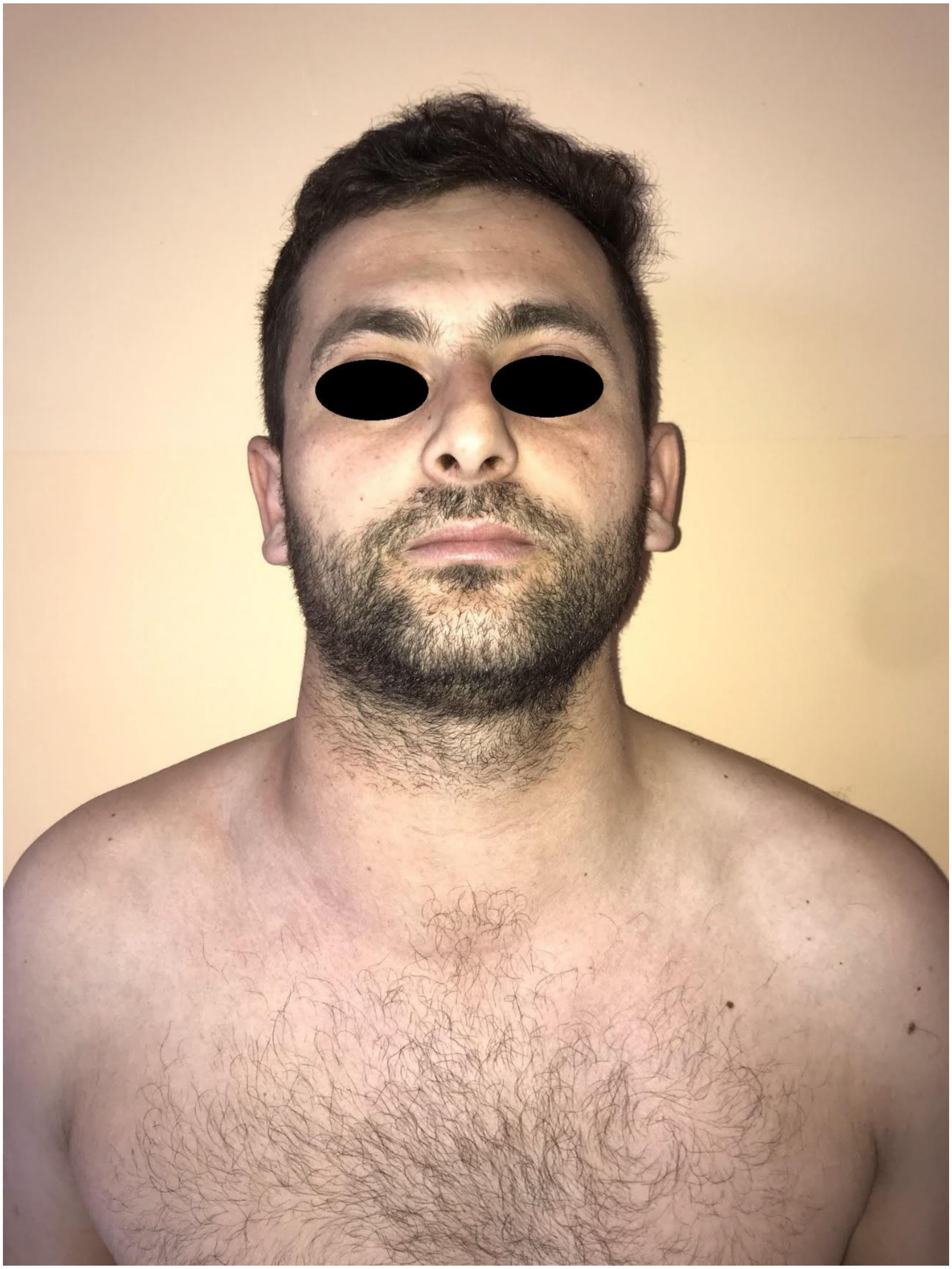

**Supplementary Figure S4. Excisional biopsy**

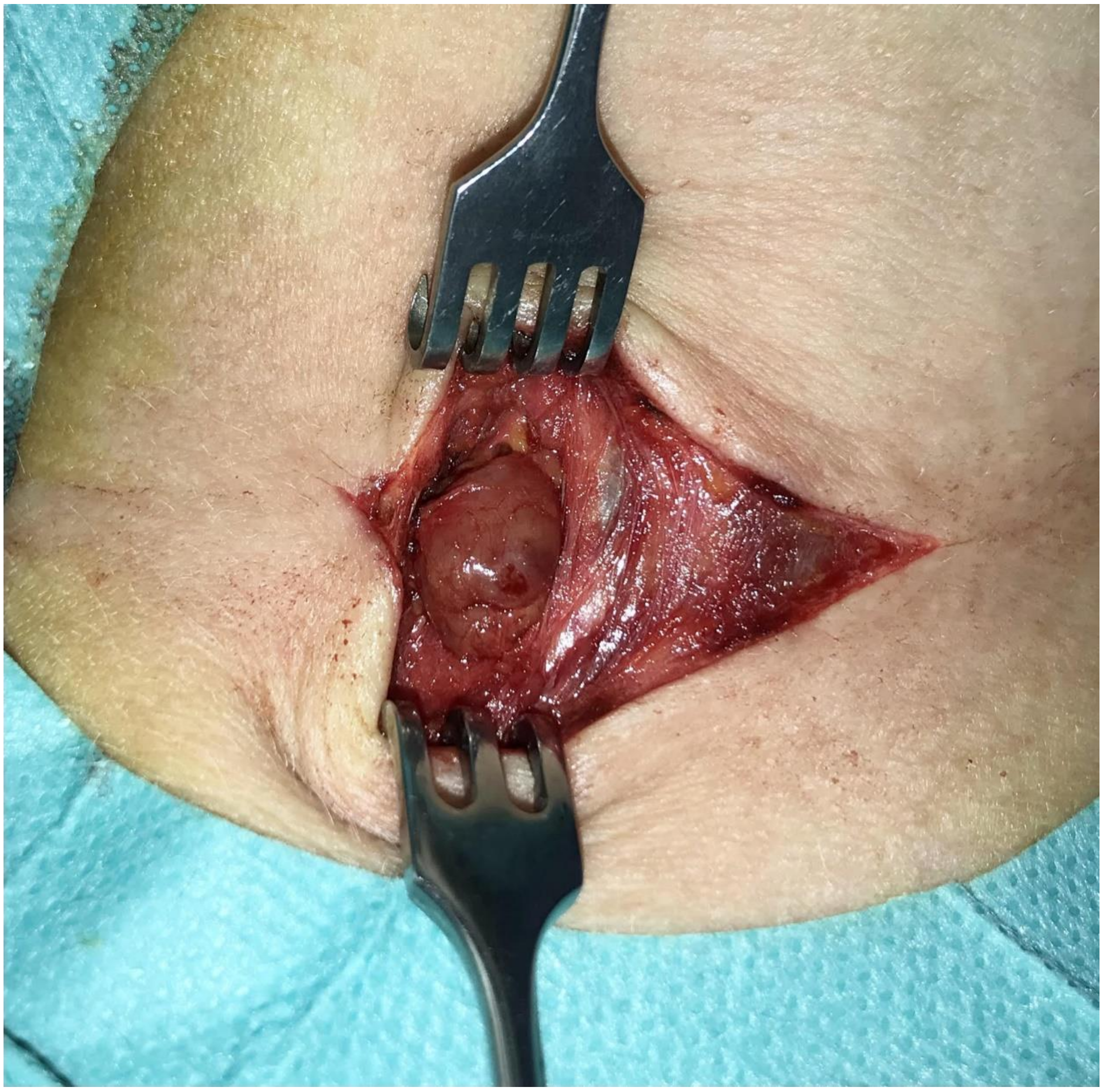

**Supplementary Figure S5. Fully excised lymph node**

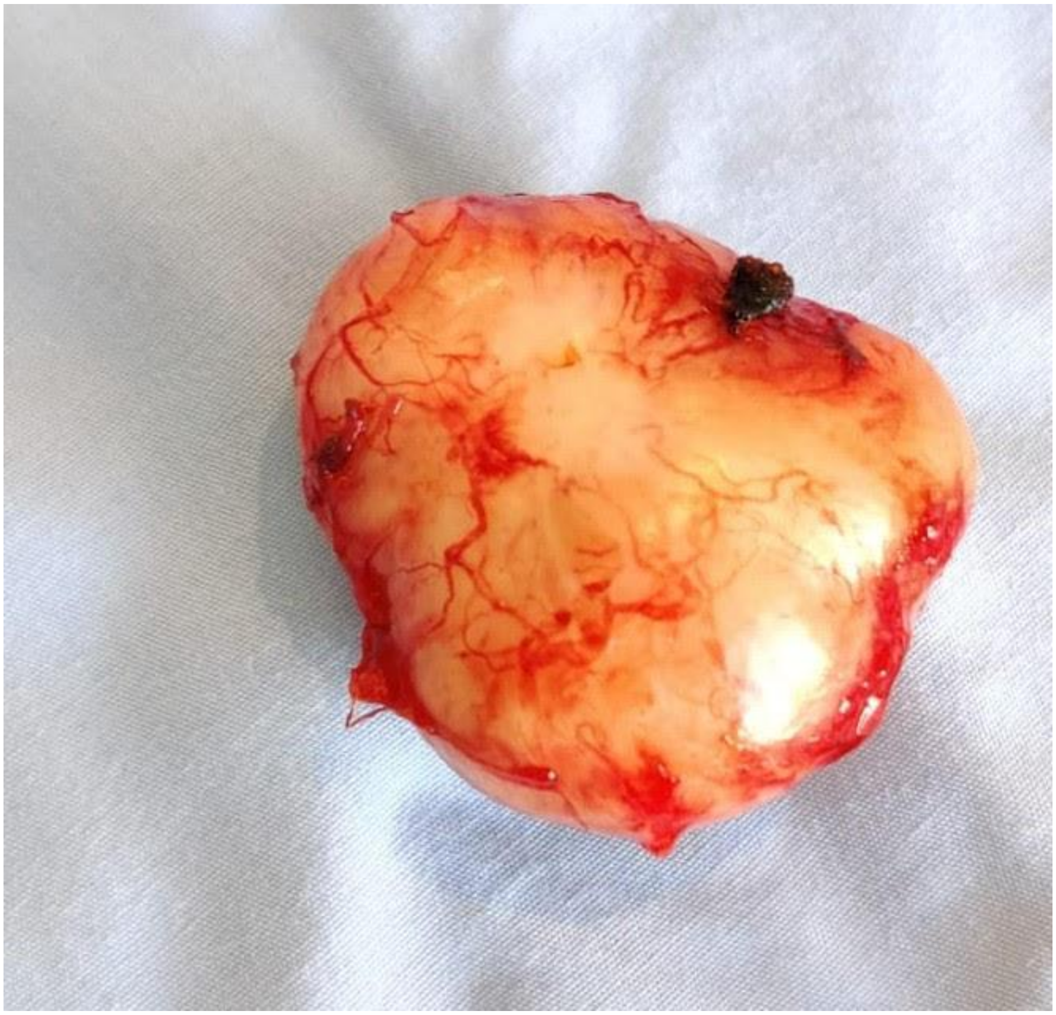

**Supplementary Figure S6. Left side neck dissection of levels Ia-IV**

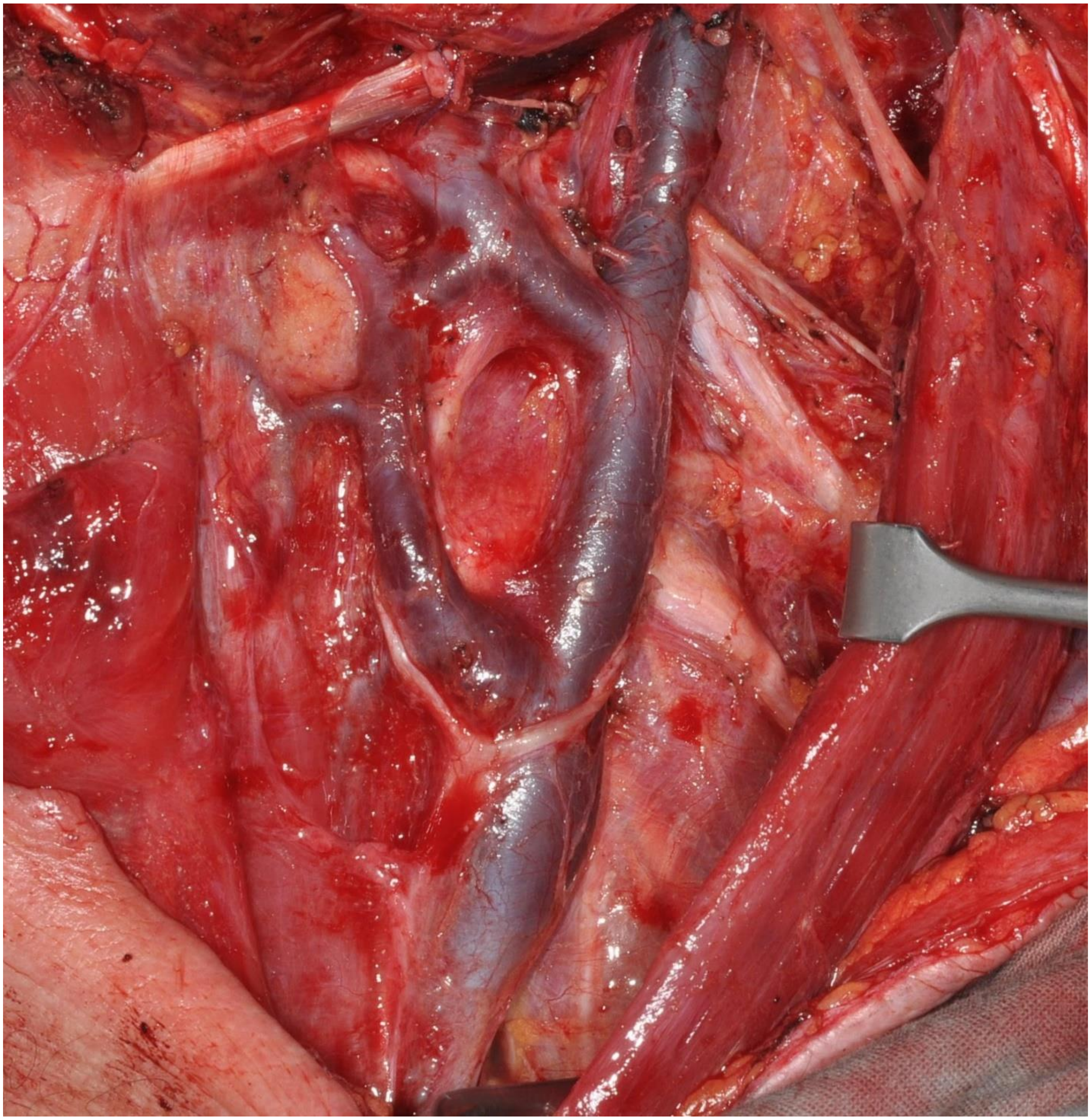

**Supplementary Figure S7. Clinical image of a patient with left side cervical LN metastasis from an oral squamous cell carcinoma.**

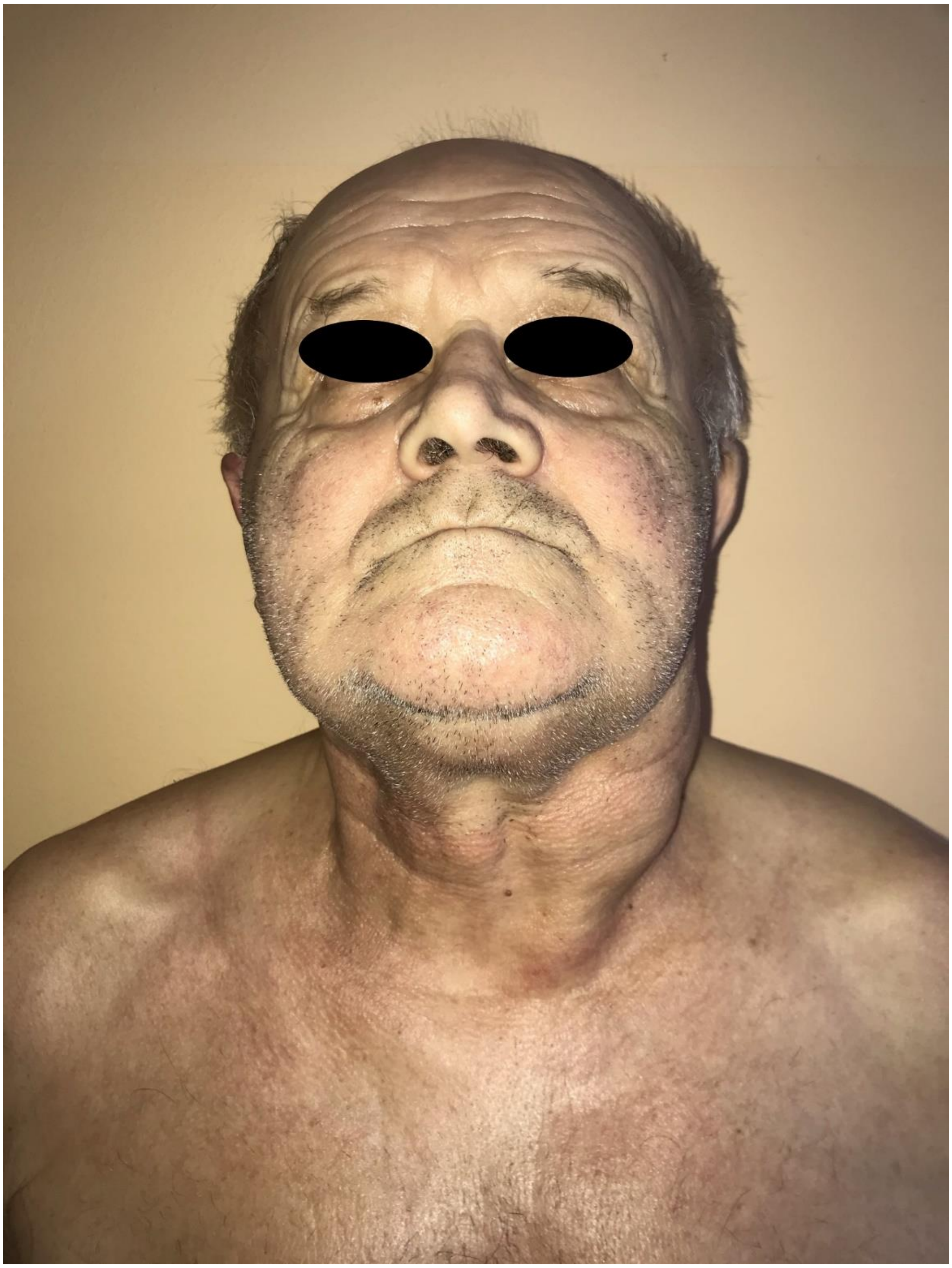

**Supplementary Figure S8. Unilateral neck dissection specimen en-block with primary tumor of the oral cavity**

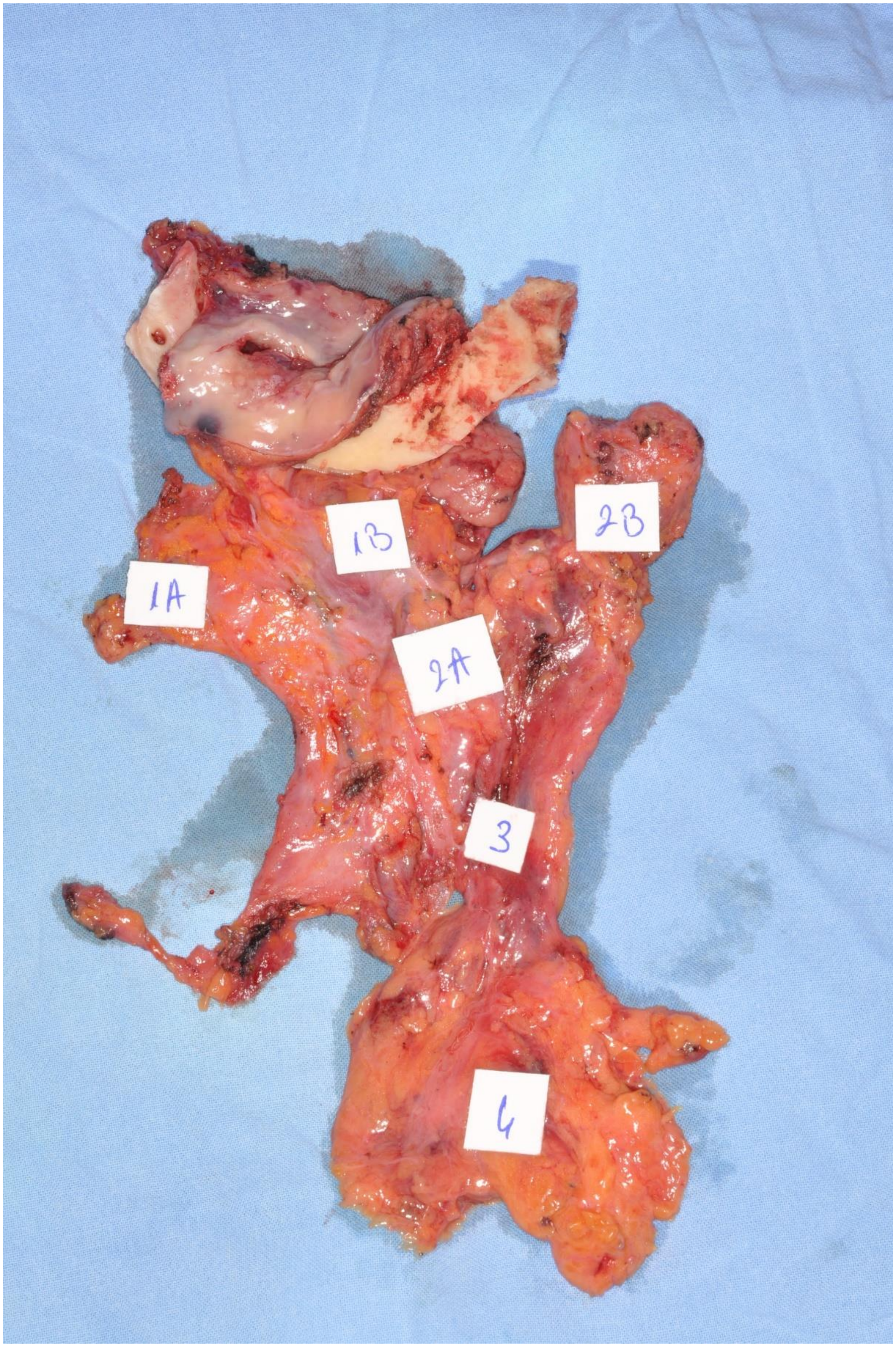

Supplement: Supplementary file 1 [file biomedicines-13-02001-s001.zip › biomedicines-3805212-supplementary.pdf]
